# Sitting on the porch choppin’ it up. HipHop and sports “go hand in hand”: a rap session with Michael Eric Dyson, PhD

**DOI:** 10.3389/fspor.2023.1232839

**Published:** 2023-08-10

**Authors:** C. Keith Harrison, Whitney Griffin

**Affiliations:** ^1^ College of Business, University of Central Florida, Orlando, FL, United States; ^2^ Psychology Department, Cerritos College, Norwalk, CA, United States

**Keywords:** HipHop culture, sport, narrative, Black American, identity, interview (conversation)

## Introduction

The canon of traditional academia has gradually expanded in the last six decades. Since the Black Arts Movement exploded in 1965, African American literature has represented a challenge to the widely accepted Eurocentric narratives taught in American universities. Situated within the Black Power Movement, the artists and intellectuals in the Black Arts Movement aligned their music, literature, drama, and visual arts with the ideologies of Black self-determination, racial pride, economic empowerment, and the creation of political and cultural institutions (1). While the Civil Rights Movement of the 1960s produced many artists and writers who focused their work on the political injustices of the time, Gates (2) specifically noted the Black Women's Literary Renaissance of the 1970s (i.e., Toni Morrison, Alice Walker, and Maya Angelou) as a key factor in broadening the validity of accepted academic narratives.

Since then, the legacy of the Black Arts Movement has made way for intellectuals in other fields to disrupt the center of traditional academia's Eurocentric narratives. In particular, as the revenue of college sports has increased, the field of sport sociology has exponentially grown to include Black perspectives. Sport sociologists have written scholarship through the prism of Black Americans to illustrate phenomena such as racial identity development (3–5), masculinity (6, 7), stereotypes (8–11), activism (12–14), academic reform (15–17), academic performance and achievement (18–21), religion (22, 23), and antideficit frameworks (24, 25). The rigorous study of sport sociology has created a space for academic credibility:

It is a thing of wonder to behold the various ways in which our specialties and the works we explicate and teach have moved, if not exactly from the margins to the center of the profession of literature, at least from defensive postures to a position of generally accepted validity (2, p. 11).

Black narratives are abundant in movements that resist racism and oppression in America. Two fields that intentionally disrupt political and cultural consciousness are sport and HipHop. To demonstrate the validity of this challenge, two scholar-activists discuss the relationship between HipHop and sport as a cultural, theoretical, and global phenomenon.

## Background

**Figure 1:**
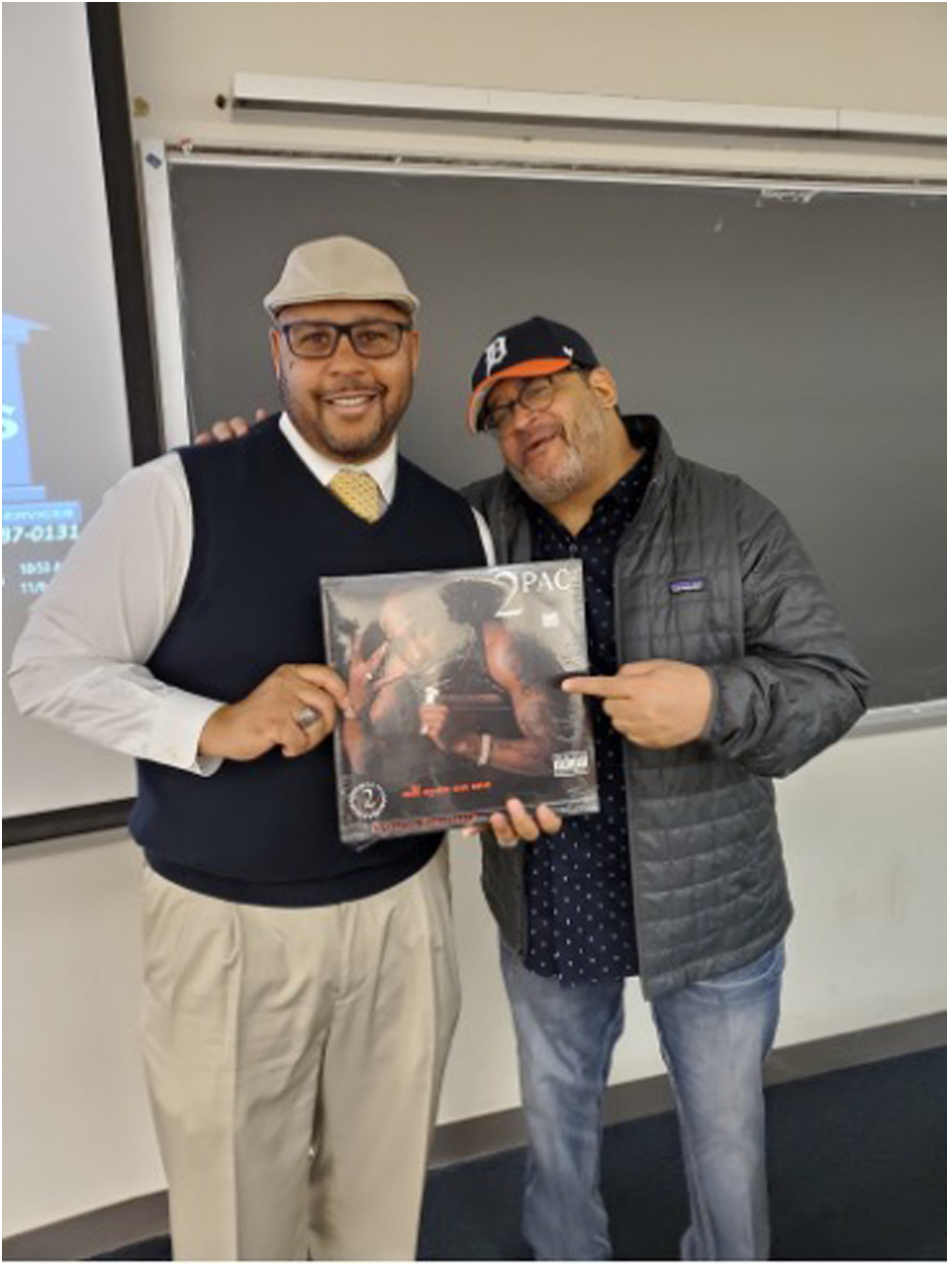
C. Keith Harrison and Michael Eric Dyson at Georgetown University near the campus at a breakfast spot before Dyson's class on HipHop in the Fall semester of 2019. The photograph is taken from the first author's collection and courtesy, circa November 2019.

Each of the Black male professors featured in this documented dialog has made major contributions to Black literature and education. Both men continue to be recognized for their distinguished innovation through awards and fellowships (see Figure 1).

Professor Keith Harrison is the founding director of the Business of Hip Hop Innovation and Creative Industries Certificate at the University of Central Florida (UCF). He has served as Associate Unit Head/Chief Academic Officer of the UCF's DeVos Sport Business Management graduate program in the College of Business and founding director (2006–2014) of The Minor That's Major™ Sport Business Management undergraduate program. He has held faculty positions at Washington State University, the University of Michigan, Arizona State University, and the UCF.

As a former scholar-baller, he played football at Cerritos College and West Texas A&M where he started as a center and graduated after making the honor roll twice. He went on to earn his graduate degree at California State University, Dominguez Hills, and his doctorate in higher education from the University of Southern California. He has published numerous peer-reviewed articles and academic books on sport sociology and HipHop in higher education. He is a senior Editor-in-Chief of the peer-reviewed *Journal of Higher Education Athletics & Innovation*, the president and co-founder of scholarballer.org, and a researcher for the National Football League's good business diversity and inclusion series. In 2021, he earned the prestigious Harvard University Hutchins Center for African and African American Research Nasir Jones HipHop Fellowship.

Professor Michael Eric Dyson is a professor, pastor, author, and media personality. He has held faculty positions at Princeton, Brown, Georgetown, and Vanderbilt, where he is a Distinguished Professor at the time of this publication. His published works span a wide range of Black culture, such as civil rights, HipHop, racism in America, and politics. With over 25 books under his name, seven have become New York Times bestsellers, including *Long time coming: reckoning with race in America, tears we cannot stop: a sermon to White America,* and *Entertaining race: performing blackness in America*. He has won such awards as the Langston Hughes Medal, American Book Award, and two NAACP Image Awards. In addition to his lectures at universities, Dyson preaches at churches around New York and Virginia.

Professor Michael Eric Dyson after his PhD at Princeton took academe, culture, and higher education by storm with his pulse for African American studies among other intellectual pursuits. In essence, he pushed the “status quo” with his passionate knowledge and unprecedented wordsmith abilities. On a personal level, his book “Between God and Gangsta Rap” was life-changing for the first author. In 1996 after his first year as a full-time faculty member at Washington State University, he attended the National Black Graduate Student Conference at Claremont, CA. During Professor Dyson's keynote, he witnessed the synergy and merger of higher education, sports, HipHop, and much more. Dyson's attire was a three-piece suit with Michael Jordan sneakers on, better known as “J's.” The point of all this is that Dr. Dyson made it cool to study HipHop in the academe, joining other pioneers such as Drs. Tricia Rose, Robin D.G. Kelley, and Cheryl L. Keyes. In short, Dr. Michael Eric Dyson made school cool, and this one-on-one interview attempts to capture his sagacity on the topic and HipHop/Sport.

## Interview transcript

**Professor Harrison:** Professor Dyson, great to see you!

**Professor Dyson:** Good to see you too, bro!

**Professor Harrison:** The first question is: Why is a book read like this on education, *HipHop and Sport: The Fifth Element* timely and possibly timeless?

**Professor Dyson**: Well, it is incredibly important, timely right now, because sports and HipHop have gone hand in hand and forging connections between young Black men especially, who are stars in one arena who identify with stars, and another. So, people who are active in the political, in the sports arena, the athletic arena identify with rappers, rappers identify with ballers, ballers want to be rappers, rappers want to be ballers right? Magic Johnson said the same thing on Arsenio Hall. So, you know when you think about this is the fifth element right? That kind of knowledge, that kind of sports, that kind of education, and the convergence of those is extremely important now because we can interpret so much of what’s going on with Black men athletically through the lens of HipHop. The ideas, the identities, the freedom, the hairstyles, the clothing style, the sartorial choices, all of that deeply imbued with a consciousness of HipHop culture and the freedom and liberty and emancipation which those styles and that appropriation give rise.

It’s timeless because constantly we have to think about that interaction, that convergence, and how it will forever impact how we know, think of, and look at young Black men. HipHop culture [is] 40 years old now maybe, but it has a deep and profound impact on expressive and athletic culture within African American society. And it is exploded exponentially beyond the borders and boundaries of Black masculinity itself. It articulates Black masculine style, desire, and ambition, but it also corrals people in from broader society into privileged circle of communication and expression among these Black men and women. And how it allows Black pop culture to be a forum to argue about, or at least engage in discussions over, the importance of Black identity. So, it is extremely timely and timeless.

**Professor Harrison:** And you’ve answered my second question Professor Dyson. I was going to ask about the intersectionality between HipHop and Sports. You’ve already hit that.

**Professor Dyson:** Thank you, sir.

**Professor Harrison:** Welcome. I co-teach a class with Reggie Saunders on the “Business of Innovation and Entrepreneurship in Sport.” You know he works at the Jordan Brand. Why is it important that two academics—my colleague and former Ph.D. student Eddie Comeaux, the first editor, me the second co-editor—and both pulled Reggie in on this project? Why is it important, Professor Dyson, to have someone like Reggie involved in academia? In a book like this.

**Professor Dyson:** Yeah well, ‘cause Reggie played and knows the game from all sides (e.g., business, culture, sports).

**Professor Harrison:** Reggie is the Jordan's right-hand man with sneakers.

**Professor Dyson**: Right exactly, well that is extremely important. Because you know people who are in the world of converse people who are in the world of creative ideas that lead to subsequent development of actual products. You know, are extremely important in helping to bridge the gulf between theory and practice. You know, how is it that ideals and ideas about Blackness, about Black masculine flare, flavor the wide receiver on the gridiron, a bit flagrant. And, you know engaging in what one would see as exaggerated behavior.

How is it that Black masculine style, in basketball with a kind of “moxie” the “chutzpah,” the kind of you know flare, the kind of charisma? Some would see it as arrogance but beautiful expressions and articulations of Black masculine style. How does that get reproduced and commodified—reproduced and commodified in a culture where products are the result of an imagination? And how does that Black imagination work? And how do we bridge the gulf between what we think of as a cultural articulation and subsequent commercial expression of that articulation? [It] is very good for brother Saunders to be there because he works on the cutting edge of trying to figure these products out and working with arguably the greatest, iconic figure within sports in the last half-century.

**Professor Harrison:** That’s a (w)rap Professor Dyson (pun intended).

## Coda

In 1996, I attended the National Black Graduate Student Conference in Claremont, CA. Professor Michael Eric Dyson gave the keynote in a suit with his “J's” [Jordan brand shoes] on. In contemporary society, sneakers are the trend and thing to wear on your 'fit regardless of how casual or dressed up one is. This was Dr. Dyson's statement of education, HipHop, and sport over 25 years ago. How educationally dope!!
